# Predicting Emotional States Using Behavioral Markers Derived From Passively Sensed Data: Data-Driven Machine Learning Approach

**DOI:** 10.2196/24465

**Published:** 2021-03-22

**Authors:** Emese Sükei, Agnes Norbury, M Mercedes Perez-Rodriguez, Pablo M Olmos, Antonio Artés

**Affiliations:** 1 Signal Theory and Communications Department Universidad Carlos III de Madrid Leganés Spain; 2 Department of Psychiatry Icahn School of Medicine at Mount Sinai New York, NY United States; 3 Gregorio Marañón Health Research Institute Madrid Spain

**Keywords:** mental health, affect, mobile health, mobile phone, digital phenotype, machine learning, Bayesian analysis, probabilistic models, personalized models

## Abstract

**Background:**

Mental health disorders affect multiple aspects of patients’ lives, including mood, cognition, and behavior. eHealth and mobile health (mHealth) technologies enable rich sets of information to be collected noninvasively, representing a promising opportunity to construct behavioral markers of mental health. Combining such data with self-reported information about psychological symptoms may provide a more comprehensive and contextualized view of a patient’s mental state than questionnaire data alone. However, mobile sensed data are usually noisy and incomplete, with significant amounts of missing observations. Therefore, recognizing the clinical potential of mHealth tools depends critically on developing methods to cope with such data issues.

**Objective:**

This study aims to present a machine learning–based approach for emotional state prediction that uses passively collected data from mobile phones and wearable devices and self-reported emotions. The proposed methods must cope with high-dimensional and heterogeneous time-series data with a large percentage of missing observations.

**Methods:**

Passively sensed behavior and self-reported emotional state data from a cohort of 943 individuals (outpatients recruited from community clinics) were available for analysis. All patients had at least 30 days’ worth of naturally occurring behavior observations, including information about physical activity, geolocation, sleep, and smartphone app use. These regularly sampled but frequently missing and heterogeneous time series were analyzed with the following probabilistic latent variable models for data averaging and feature extraction: mixture model (MM) and hidden Markov model (HMM). The extracted features were then combined with a classifier to predict emotional state. A variety of classical machine learning methods and recurrent neural networks were compared. Finally, a personalized Bayesian model was proposed to improve performance by considering the individual differences in the data and applying a different classifier bias term for each patient.

**Results:**

Probabilistic generative models proved to be good preprocessing and feature extractor tools for data with large percentages of missing observations. Models that took into account the posterior probabilities of the MM and HMM latent states outperformed those that did not by more than 20%, suggesting that the underlying behavioral patterns identified were meaningful for individuals’ overall emotional state. The best performing generalized models achieved a 0.81 area under the curve of the receiver operating characteristic and 0.71 area under the precision-recall curve when predicting self-reported emotional valence from behavior in held-out test data. Moreover, the proposed personalized models demonstrated that accounting for individual differences through a simple hierarchical model can substantially improve emotional state prediction performance without relying on previous days’ data.

**Conclusions:**

These findings demonstrate the feasibility of designing machine learning models for predicting emotional states from mobile sensing data capable of dealing with heterogeneous data with large numbers of missing observations. Such models may represent valuable tools for clinicians to monitor patients’ mood states.

## Introduction

### Passively Sensing Behavioral Biomarkers

The subjective experience of mood is one of the most valuable sources of information about an individual’s mental health [1]. Self-reported mood is a critical component of the mental status exam interview, which is to psychiatry what the physical exam is to other fields of medicine [2]. Furthermore, clinicians routinely ask questions about mood during clinical encounters. The presence of a specific mood state is a required criterion for many psychiatric diagnoses according to the Diagnostic and Statistical Manual of Mental Disorders, fifth edition (eg, depressed mood to diagnose a major depressive episode; elevated, expansive, or irritable moods to diagnose a manic episode; etc). Mood is a predictor of psychiatric outcomes, and mood changes can be a harbinger for psychiatric decompensations. Therefore, accurate monitoring of mood states is a crucial component of mental health care. For example, both valences of mood states [3] and their variability [4] have been shown to predict important outcomes, such as several binge-eating episodes in bulimia nervosa [4] and treatment adherence in patients with bipolar disorder and opioid use disorders [3,5].

Until recently, information about mood was only available to clinicians by directly questioning patients in person, either over the phone or via telepsychiatry video platforms. However, technological advances over the last few decades have allowed for real-time monitoring of patients’ self-reported mood states. Smartphone-delivered ecological momentary assessment (EMA), also known as experience sampling, “assesses individuals’ current experiences, behaviors, and moods as they occur in real-time and in their real-world settings” [6]. However, despite these technological advances, this form of mood state assessment relies on an individual’s current level of insight and willingness and ability to interact with the EMA platform. Many psychiatric disorders cause behavioral changes that may decrease an individual’s likelihood of interacting with an EMA tool (demotivation, apathy, and survey fatigue), causing missing data, not at random. Therefore, identifying objective behavioral biomarkers of mood states that can be passively sensed without patient participation is a research priority.

Through patients’ mobile phones and other wearable devices, continuous sensor data can be collected in a noninvasive manner, providing valuable information about everyday activity patterns. The possibility of inferring emotional states by analyzing smartphone use data [7-9], GPS traces of movement [10,11], social media data [12], and even sound recordings [13,14] has become a growing research focus over the past decade. Such approaches can be used to analyze individuals’ emotional patterns, enabling the better self-management of one’s activities and behavioral choices. Moreover, for patients with mental illnesses and their caregivers and health care providers, these models could provide a means to predict mental health crises and maladaptive behavioral patterns and allow for early intervention.

### Related Work

In the past few years, numerous studies have demonstrated the potential of exploiting mobile sensing data to infer users’ emotional states and well-being. In an older study, LiKamWa et al [7] developed MoodScope, a statistical inference model for predicting the users’ daily mood average based on the circumplex mood model [15,16], from communication history and app use patterns. They collected data from 32 participants over 2 months and reported an initial accuracy of 66%, which improved over time for personalized models.

Jaques et al [17] conducted a study using physiological signals, location, smartphone logs, and survey responses collected over a month from 206 college students to model students’ happiness. They applied classical machine learning methods, such as support vector machines (SVMs), random forests (RFs), neural networks, logistic regression (LR), k-nearest neighbor, naive Bayes, and Adaboost, to perform the classification task and reported 70% accuracy.

Another study focusing on predicting college students’ stress and mental health status was conducted by Sano et al [18]. They compared lasso regression and SVM with linear and radial basis function kernels for 2 classification tasks: low or high stress and low or high mental health categories. They reported over 70% accuracy and showed a significant performance increase when data from wearable sensors (such as skin conductance and temperature) were used, compared with behavioral data derived from phone sensing.

Umematsu et al [19] compared nontemporal (SVM and LR) and temporal (long short-term memory [LSTM]) machine learning methods to forecast the stress level of the upcoming day using a predefined number of days of previous data (physiological signals, mobile phone use, location, and behavioral surveys). A more recent study by Morshed et al [20], who used the StudentLife [21] and Tesserae [22] data sets, demonstrated that mood instabilities (computed from the mapping of moods on the photographic affect meter scale [23] to arousal and valence values) are predictable from features derived from passive sensor measurements.

In a large-scale study conducted by Servia-Rodríguez et al [24], the researchers used passive sensing data and self-reported moods collected for about 3 years from 18,000 users to build a predictive model for users’ mood. They trained a deep neural network of stacked restricted Boltzmann machines for a 2-class classification problem (positive and negative mood). They reported above 60% prediction accuracy for weekdays and 70% for weekends.

An LSTM recurrent neural network (RNN)–based analysis, performed by Suhara et al [25], showed that applying a temporal model for forecasting severe depressive states outperformed nontemporal models. Their study relied on a large-scale longitudinal data set of self-reported information about mood, activity, and sleep of 2382 self-declared depressed people over 22 months.

Cho et al [26] conducted a prospective observational cohort study to evaluate the mood of 55 patients with major depressive disorder and bipolar disorder types 1 and 2. They collected light exposure data passively via mobile phones of patients and self-reported daily mood scores. Using activity trackers, they registered activity, sleep, and heart rate data. This information was then processed into 130 features based on circadian rhythms, and mood prediction was performed using the RF method. Their approach generally showed good sensitivity and specificity for mood state and episode prediction.

Taylor et al [27] focused on building personalized models for forecasting the next day’s mood (good or bad), health (fair or poor), and stress intensity (low or high). The multitask learning-based approach used data about the physiology and behavior of 206 undergraduate students and the weather of the current day, collected for 30 days. Their results showed that tomorrow’s well-being could be predicted with 78% to 82% accuracy using a personalized model based on the present day’s data. Busk et al [28] proposed a hierarchical Bayesian approach for forecasting mood for up to 7 days from smartphone self-assessments of 84 patients diagnosed with bipolar disorder. Their best performing model used a history of 4 days of self-assessment, indicating that short-term historical mood is a significant predictor.

Another recent observational study by Darvariu et al [29] combined user-reported emotional information, passive sensing data, and visual context information from individuals’ surroundings in the form of images to develop deep learning techniques for emotional state inference. Their findings showed context-dependent associations between self-reported emotional states and the objects surrounding the individuals.

These studies provide insight into the potential of using mobile sensor data to infer individuals’ mental well-being. However, none of these studies reported working with a data set consisting of observations from a nonexperimental setting or dealing with large amounts of missing data. Moreover, in most of these studies, the problem they are trying to solve is a 2-class classification problem. Here, the problem is approached from a more refined perspective (ie, predicting emotional state in terms of both valence and arousal dimensions).

### Objectives

This study focuses on applying machine learning algorithms to predict mood states based on passively sensed behavioral patterns. Specifically, we aim to assess which behavioral features provide the most important information about daily emotional valence. The study was conducted by using data collected via a clinically validated eHealth platform (eB2 MindCare) [30,31]. This app is designed to run unobtrusively in the background of an individual’s smartphone. It automatically and continuously gathers information about behavior, collected via both the individual’s smartphone and wearable devices. It also provides an electronic diary type interface for users to register information about their emotions and other important events.

## Methods

### Participants

The data for this experiment were collected via eB2 MindCare [32] in collaboration with public mental health hospitals Hospital Universitario Fundación Jiménez Díaz and Hospital Universitario Rey Juan Carlos, Madrid, from clinical outpatients [30,33,34] and nonpathological volunteers from Universidad Carlos III de Madrid and Universidad Católica de Valencia. Patients were invited to participate in the data collection process by their clinicians. The research followed the code of ethics defined in the Declaration of Helsinki by the World Medical Association.

### Patient Inclusion and Exclusion Criteria

Patients were included in the study if they were at least 18 years old clinical outpatients diagnosed by specialists at the institutions mentioned above with mental disorders or were attending therapy groups (such as support groups for cyberbullying and relaxation) at these institutes. They had to own a smartphone running on Android or iOS operating systems, which they connected to a Wi-Fi network at least more than once per week. Only patients who provided written informed consent for the eB2 study were included. None of the patients were paid for participating in the study.

### Data

The eB2 MindCare app collects data from different sources (the mobile phone’s sensors, Google Fit, and wearables such as Fitbit and Garmin) at different time intervals. After installing the app, the users are taken through an onboarding phase, in which they are asked to give permission for specific data collection streams, depending on the operating system. In addition to passively collected data, users can record information about their experiences, quality of sleep, and emotional state during the day. The app offers the following emotion options to choose from: angry, disgusted, scared, sad, overwhelmed, tired, grief, neutral, relaxed, motivated, happy, and delighted. Within a day, patients may register their emotions multiple times.

Daily summary values of 6 passively collected observations were considered: step count, distance traveled, hours of sleep, hours of phone use, time spent at home, and the number of locations visited. An additional binary variable was included, indicating whether the patient practiced sports during the day. The step count is recorded every 5 minutes, and the daily summary value corresponded to the sum of the registered entries. App use information was gained similarly. Distance information is gathered every minute, whereas location data are gathered at 5-minute intervals. Locations are obfuscated with an offset and randomly rotated to protect users’ data. From these sources, the daily travel distance and the number of visited locations were computed. Time spent at home was computed using clustering based on the most common user locations throughout the day. There is a hierarchical set up for hours of sleep for the credibility of different sources; if data are manually introduced by the user or calculated by the phone but confirmed by the user, that value is first considered. Otherwise, the following ordering holds: sleep data by iOS, sleep data by Garmin, sleep data by Fitbit, sleep data calculated from light, app use and steps data, and sleep data calculated by the phone. The devices register sport-related activities on change, and the daily summary encompasses the total number of times each action was performed.

A subset of 943 users (patients and nonpathological subjects) was selected with at least 30 days of passively sensed data in the eB2 database between January 2019 and March 2020. The number of recorded days per patient varied from 30 to 487 with a mean of 190 (SD 122). Demographic information was available only for 871 users. All the users were Spaniards. Of these, 63.5% (553/871) were female and 25.1% (219/871) were male, and gender information was not available for the remaining 11.4% (99/871). All age groups were adequately represented in the data set, with a mean age of 41 years (range 18-77 years) computed at the beginning of the measurement period. The patient population came from 2 main categories: 61.3% (534/871) were outpatients from external psychiatric consultancy and 22.1% (192/871) were suicidal high-risk outpatients. The remaining 16.6% (145/871) were nonpathological users. Note that neither demographic nor diagnostic information was used in the rest of the study.

A well-known framework for dealing with emotional experience characterizes emotions in a 2-dimensional space defined by Russel [15,16]. The arousal and valence are combined, with valences ranging from highly negative to highly positive and arousal ranging from low to high. Daily emotional valence and arousal metrics were determined using raw emotion data entered by patients. Valence was then computed as the sign of the difference between positive and negative emotion counts, whereas arousal was determined based on the categories in the study by Scherer [35].

**Figure 1:**
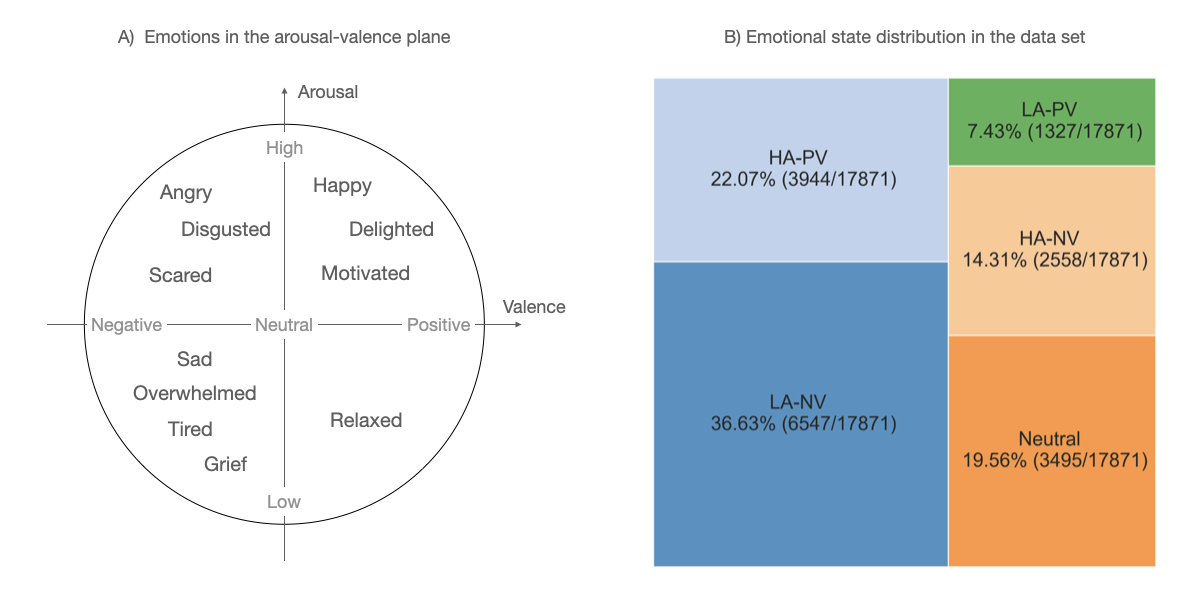
Projection of emotions into the arousal-valence plane and their distribution in the data set. HA-NV: high arousal-negative valence; HA-PV: high arousal-positive valence; LA-NV: low arousal-negative valence; LA-PV: low arousal-positive valence.

The left subfigure in Figure 1 shows the projection of emotions to the arousal-valence plane. The emotions listed on the graph are those that patients can register via the eB2 app. As the right subfigure in Figure 1 shows, there is a significant imbalance between the different emotional labels. The majority correspond to negative emotional valence (9105 entries), followed by positive emotions (5271 entries) and only 3495 neutral entries in the entire data set. Moreover, as emotions are self-reported, with users not being prompted in any way to fill in this information, these entries are scarce compared with passively sensed behavioral data.

As data have been collected from several sources and received in different formats, the raw daily summary data have many anomalies and unwanted information, and hence, noise. The presence of noise in the data can degrade the performance of machine learning methods. Therefore, it is important to preprocess the data before using it as an input to any machine learning algorithm. The first step of preprocessing was removing any negative values, followed by thresholding the time-related variables to 24 hours, the step count to 30,000 steps per day, and the distance to 500 km. Data were then standardized over all patient sequences, making each input feature 0 mean (SD 1).

Moreover, the data set contained a large percentage of missing observations. Figure S1 in Multimedia Appendix 1 shows the missing pattern in the entire data set. Approximately 84% (150,615/179,740) of the observations were partial, a bit over 5% (9399/179,740) were complete, and the remaining 10% (19,726/179,740) were entirely missing. Slightly less than 10% (17,871/179,740) of the observations were labeled by an emotion entry. A total of 271 patient sequences were observed for all 7 summary variables. Close to half of them did not have information about the time spent at home and the number of locations visited. The app use information was also completely missing for 226 patients. In addition, 114 patients had more than 30 consecutive days of completely missed observations (range 31-372).

### Probabilistic Generative Models for Dealing With Missing Data

Imputing missing data using statistical measures such as the mean, median, or even interpolation fails when the percentage of missing data is very high. These approaches can reduce variability in the data set and introduce bias. However, probabilistic generative models can learn the underlying distributions in a data set by adjusting the model parameters to best account for the data in the sense of maximizing the evidence, even in the presence of missing data. Mixture models (MMs) [36] and hidden Markov models (HMMs) [37] are frequently used types of such models.

MMs comprise a finite or infinite number of components, possibly different distributional types, that can describe different data features. The data can then be modeled in terms of a mixture of several components, where each component has a simple parametric form (such as a Gaussian). The model is formulated in terms of latent variables, which represent the component each data point was sampled from and learned from the observed features, referred to as observables by adjusting the model parameters, which define the observable emission probabilities, such that the MM best accounts for the data in the sense of maximizing the evidence.

HMMs are temporal MMs that are commonly used for time-series analysis. These are generative models characterized by a set of observable sequences. The discrete states of the HMM are assumed to have been generated by a first-order Markov chain process, and each observation depends only on the paired state. An HMM comprises an initial state probability distribution, a state transition probability distribution, and a symbol emission probability distribution. Both MMs and HMMs were trained using the expectation-maximization algorithm.

In this study, the observed data were heterogeneous. Practice sport and emotional state are categorical, and the rest of the variables are assumed to be real-valued. Both MMs and HMMs can deal with missing data, without requiring imputation before training, via marginalization. For the Gaussian parameters, the diagonal covariance matrices were considered. Furthermore, both generative models were trained in a semisupervised manner for emotional valence and arousal-valence discrete observations. Namely, the different emotional states’ emission probabilities were fixed for some of the components, whereas others were adjusted during training, such as the other model parameters. For instance, in a 5-component MM with binary label emissions, the emission probability for label 0 of the 3 components can be set to 1, forcing the components always to emit label 0. In contrast, the other 2 components can always be forced to emit label 1.

### Emotion Prediction Models

A series of experiments were conducted for emotional status prediction accuracy using both nontemporal and temporal machine learning models. The underlying motivation was to analyze whether there were long-term dependencies in the data concerning patients’ daily emotional states.

Probabilistic generative models (MM and HMM) were used to perform the imputation. Note that only the input features were imputed, and the emotion labels were not. When using MMs, first, for each observation, the posterior distribution needs to be inferred to find which component the observation is most likely to belong to; then, the missing attributes are imputed by a sample generated from that component. Information about the emotional state belonging to the current observation was not included in the posterior computation (otherwise, the model would overfit). When using HMMs, all observation sequences were first decoded using the Viterbi algorithm on the trained HMM. This method finds the most likely sequence of components that could have resulted in the given observation sequence. Once the state sequence was determined, the missing data were imputed by the samples generated from the corresponding states for each time step. The state posterior probabilities were computed by applying the forward algorithm [37], leaving out the current emotional observation.

For nontemporal machine learning methods, LR, support vector classifier, random forest classifier (RFC), and multilayer perceptron (MLP) were considered. These models allow comparisons with previous emotional state studies [16-19,26]. A grid search was performed for each case for hyperparameter tuning.

RNNs [38] have recurring inputs to the hidden layer; this allows them to remember input states from previous time steps, which can carry important information for future time-step predictions. There are 3 common types of RNNs: vanilla RNN, LSTM [39], and gated recurrent units (GRUs) [40]. Vanilla RNNs have short-term memory. If the observation sequence is rather long, these models have difficulty remembering relevant information from earlier time steps. LSTM and GRU cells, which contained gates that regulate the information flow, were designed to solve this problem.

In this experiment, RNNs of each of the 3 types were tested. A single layer with 64 hidden units was used, whose output was connected to a dense layer. Finally, the softmax activation function provides the predictions. The model was trained using the Adam method and the negative-log-likelihood loss for 50 epochs, using early stopping. One-layer RNNs with vanilla RNN, LSTM, and GRU cells were trained using 64 hidden units for each case. More complex models, such as dilated RNN, multilayer RNN, and temporal convolutional networks, have also been tried. However, they did not improve performance, proving that simpler RNNs could explain the data’s temporal correlations.

### Personalized Models

To improve the above models, hierarchical Bayesian regression models were proposed to account for individual differences and predict the emotional state of patients. The proposed model allows intercepts to vary across patients, according to a random effect, while having a fixed slope for the predictor (ie, all patients will have the same slope). In our model for individual *j*, observation *i*, target variable *y_ji_*, and input features *x_ji_*:

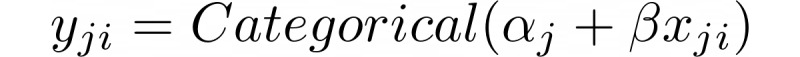



where the random intercept effect is drawn from the population distribution:

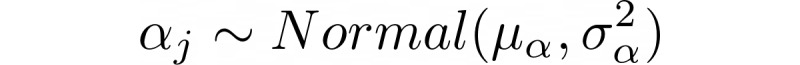



The population mean and SD are independent normal and half-normal priors. By setting a separate bias term for each patient, rather than fitting separate regression models for each patient, multilevel modeling shares strength among patients, allowing for a more reasonable inference in patients with little data. The models were trained with Stein Variational Gradient Descent [41,42] for 50 epochs using the Adam optimizer.

### Evaluation and Interpretability

Accuracy, area under the receiver operating characteristics curve (AUC-ROC), and area under the precision-recall curve (AUC-PRC) were used as the evaluation metrics. AUC-ROC is commonly used for both balanced and imbalanced classification problems because it is not biased toward the majority or minority class. However, AUC-PRC scores provide more insight into the minority class when the problem is very skewed. As the AUC-ROC and AUC-PRC scores are computed for binary classification problems, in the case of multiclass targets, different types of averaging can be performed on the data. The reported results were microaveraged, meaning that the metrics are global, computed by counting the total number of true positives, false negatives, and false positives.

On the basis of several model interpretability methods, Lundberg and Lee [43] defined the Shapley additive explanations (SHAP) value, a modality to explain any machine learning model’s output. The SHAP values can provide global interpretability to the machine learning models by showing how much each feature contributes, positively or negatively, to the target variable. This approach was used in this study to analyze the feature importance for the models. Moreover, this method can be applied to analyze the decisions for individual predictions, which provides better insights into the relationships between passively collected mobile data and self-reported emotions.

### Experiments

For MM and HMM training, only those patient sequences with at least partial observations for each of the 7 features and emotions were used. Moreover, the maximum sequence length was limited to 365 days, and sequences that had more than 30 days of consecutive missing data for all variables were discarded. After this elimination process, 233 sequences were used to train both the MMs and HMMs with different numbers of states. These patient sequences were excluded from both the training and test sets of the later models.

For the global models, the data set containing the remaining 710 patient sequences was divided into training and test sets using 80% of the sequences for training and 20% for testing. These data sets were kept independent. The train-test split cannot be done for the personalized models by randomly selecting a given percentage of the patients for training while leaving the others for testing, but all 710 patients must be included. Therefore, the patient sequences themselves were split into training and test sections. The first 80% of the labeled observations, in chronological order, were used for training, and the remaining samples were used for testing.

As the LR, support vector classifier, RFC, and MLP cannot directly exploit time-series data, we created the following 2 cases as inputs for these models. First, the input-output pairs consisted of 1 day of labeled observation. Second, 3 days and a week before the entered emotion was considered and concatenated into a single feature vector. In the case of the temporal models, training was performed with 30-day, 3-month, and 6-month long sequences.

Before creating the above feature vectors, the missing data in each patient sequence were imputed by the MM or HMM samples. For models trained with mini-batch stochastic gradient descent, every data point is imputed every time it enters the optimizer. The sequences were decoded multiple times, and missing data were imputed by samples generated from the corresponding state.

We designed two types of experiments. The first type is limited to the projection of the recorded emotions to a single axis of the arousal-valence plane. The second set of experiments considered 2-dimensional projections. A total of 3 different settings were analyzed for the classifiers’ input features, as follows: using the imputed raw data, using the MM or HMM posterior probabilities instead of the raw input features, and using the raw inputs concatenated with the MM or HMM posterior probabilities.

## Results

### Generative Models

**Figure 2:**
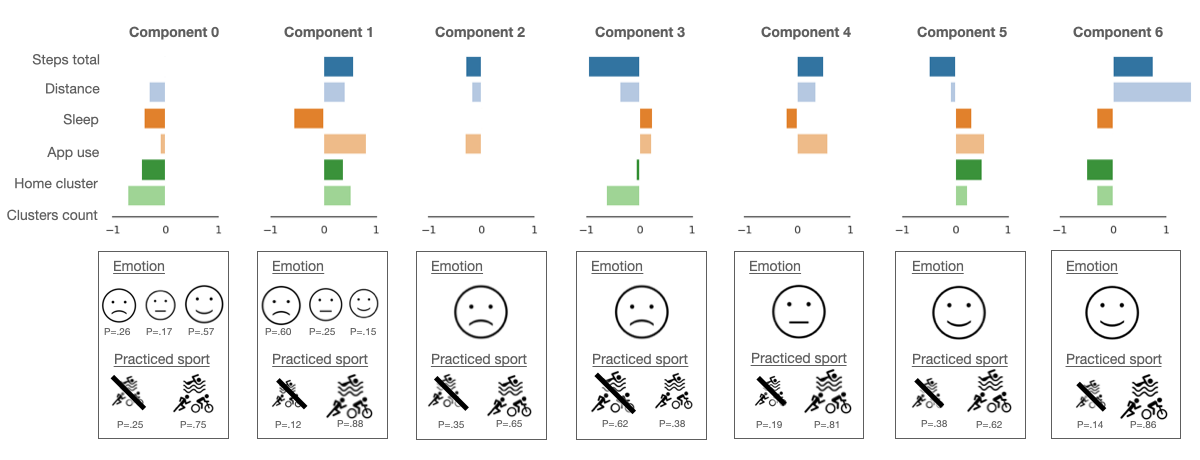
The 7-component mixture model structure was used for emotional valence modeling with each Gaussian mean in each component and indicating discrete emission probabilities. The size of the icons indicates the magnitude of the discrete emission probabilities (emotion and sport). In terms of features, "steps total" refers to step count, "distance" refers to the distance traveled, "sleep" refers to the hours of sleep, "app use" refers to the hours spent using different apps, "home cluster" refers to the time spent at home, "clusters count" refers to the number of visited locations, and "practiced sport" is an indicator of whether the patient practiced any sports. Of note, the negative mean values were a result of the normalization of the features.

After experimenting with several hidden state setups, 7 hidden components captured the data’s underlying patterns well, leading to the best results when a classifier was applied to the data later to predict emotions and provide interpretable states. In this case, the emission probabilities of the five states were fixed so that two pairs of states always emitted negative and positive emotions, and one always emitted a neutral emotion. The different components turned out to be specialized, as they captured contrasting behaviors (Figure 2).

Focusing on the three components that mainly emit negative emotional valence (components 1, 2, and 3), it can be seen that the corresponding modeled behaviors are contrasting. Component 1 represents days when the patients are quite active, visit multiple locations, spend a significant amount of time using their phones, and sleep very few hours. Component 2 is characterized by fewer steps and low app use. Component 3, however, captures days with low activity and mostly spent at home. The corresponding sport-related discrete emissions show that the patients practice some sport (>15 minutes of walking, biking, running, other, or a combination of those) in components 1 and 2, but less likely in component 3. Components 0, 5, and 6 correspond to positive emotional valence. They also seem to capture significantly different behavioral patterns. In component 0, the patients seemed to sleep less and did not spend much time at home; component 5 captured days with more time spent at home and excessive phone use. Component 6 captures the days of travel. Finally, the component capturing neutral emotions indicates days with medium activity and more app use.

**Figure 3:**
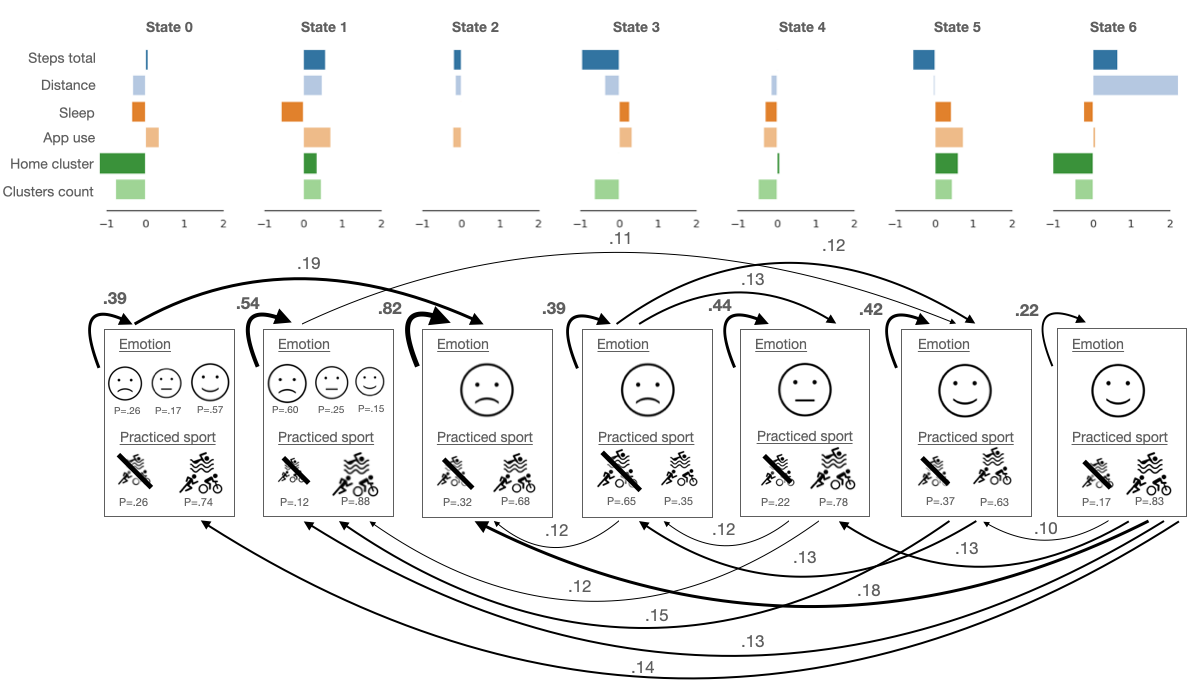
The 7-component hidden Markov model structure was used for emotional valence modeling with each Gaussian mean in each component and indicating discrete emission probabilities. The size of the icons indicates the magnitude of the discrete emission probabilities (emotion and sport). Only the transitions with a higher than 0.1 probability are shown in the graph. In terms of features, "steps total" refers to step count, "distance" refers to the distance traveled, "sleep" refers to the hours of sleep, "app use" refers to the hours spent using different apps, "home cluster" refers to the time spent at home, "clusters count" refers to the number of visited locations, and "practiced sport" is an indicator of whether the patient practiced any sports. Of note, the negative mean values were a result of the normalization of the features.

Including the temporal properties of HMMs, the trained generative model with 7 hidden states and the same fixed emotional state emissions led to very similar interpretable outcomes as the MM (Figure 3). The temporal characteristics were not very strong. States 2 (with fixed negative emotional valence emission) and 1 (with mainly negative emotional valence emission) had the highest self-transition probabilities. If the self-transition probabilities are large, it indicates a stable state. States 0, 3, 4, and 5 have somewhat large self-transition probabilities, which suggests that days with positive and negative but also neutral emotions following each other are common in the patient population.

In the arousal-valence case, the 7-state generative models had 1 state assigned to all the emotional state emissions, and the other 2 were trained with the rest of the parameters. Similarly, as before, the states appear to capture specific behaviors, such as days of medium activity but mostly spent at home, more active days, days with more travel, and so on (Figures S2 and S3 in Multimedia Appendix 1 provide the sketches of the 7-component MM and HMM, respectively).

### Predicting Emotional Valence

Figure S1 in Multimedia Appendix 2 compares the accuracy and the microaverage AUC-ROC and AUC-PRC scores for the trained classifiers in the 3 experimental set ups, as described in the *Experiments* section. Most classifiers achieved significantly higher performance than random guessing (AUC-ROC=0.5). As the figure shows, the models perform the worst on the raw data. Using the HMM or MM posteriors as input features or combining the raw data with the posteriors increases the performance. Table 1 compares the best performing models when using the MM and HMM posteriors. The difference in the results obtained with the MM posteriors and HMM posteriors is minimal. This indicates that the temporal dimension is not very relevant to the problem at hand; hence, a simpler generative model is sufficient for the problem.

**Table 1:** Performance comparison of the best performing models using mixture model and hidden Markov model posteriors as classifier input features.

Model and classifier input features		Accuracy (%)	Area under the receiver operating characteristics curve	Area under the precision-recall curve
**Multilayer perceptron using 7 days of observations as input features**				
	Mixture model posteriors	65	0.81	0.70
	Hidden Markov model posteriors	64	0.80	0.69

The best performing model was the MLP with the posteriors of 7 days of observations as input features. Concatenating the posterior probabilities for 3 days or 7 days of observations significantly improves the performance; however, training RNNs with longer observation sequences leads to decreased performance. This suggests no substantial seasonality or long-term trend of the self-reported emotions; thus, time-series models are not needed for the emotional state prediction task.

Generally, the most misclassified emotional state is the neutral state (refer to Table S1 in Multimedia Appendix 2 for confusion matrices). In most cases, it is confused with a negative emotional state and reasonably often with a positive one. There is some confusion between positive and negative emotional states, but somewhat fewer for negative emotions. This suggests that the models are more sensitive to detecting negative emotions, which can be desirable; for example, if the app’s goal is to detect periods when the patient is feeling down.

### Predicting Emotional Arousal Valence

In the second experiment, the target variables were the emotion projections into the 2-dimensional arousal-valence space, based on the categories in the study by Scherer [35]. Hence, the problem becomes a 5-class classification task. Here, we aimed to test the possibility of predicting daily emotions on a finer scale than the 2-class valence analysis presented above.

The best performance for the emotional arousal-valence prediction, with 48% accuracy (compared with the baseline of 20%), 0.77 AUC-ROC, and 0.50 AUC-PRC, was obtained by the RFC with 7 days of data concatenated with the MM posteriors. The GRU network trained on 30-day sequences reached results closest to those from the temporal models: 42% accuracy, 0.69 AUC-ROC, and 0.36 AUC-PRC. In this setting, the added MM posteriors’ effect was more significant than the emotional valence prediction case. Using the posteriors as input features led to a 23% performance increase in some of the models. Table S2 in Multimedia Appendix 2 provides a detailed performance comparison of the models.

Predicting more refined emotional states is a difficult task, as not only are there more classes to distinguish, but the class imbalance is also more accentuated. The trained models became somewhat biased toward the majority classes, resulting in the wrong classification of the minority classes (high arousal-positive valence and low arousal-positive valence). Generally, when the predictor variable is well separable, and there are no overlaps between the different classes, this separation can compensate for the imbalance; however, in this data set, that is not the case. Standard techniques to combat the imbalance problem, such as upsampling of minority classes, downsampling of majority classes, and one-versus-rest training, were applied; however, these only led to a slight improvement. Therefore, these results have not been reported.

### Personalized Models

The previously presented models try to explain the variability of the observations by considering the patient population. As shown before, these models do not provide enough diversity when the classifier takes as input 1-day worth of data. Personalized models can provide a more scalable and accurate way to achieve better representations for individual patients.

The posterior probabilities obtained from the MM components were used as input features for the personalized models because they proved to improve the prediction outputs of earlier experiments. In the global models presented previously, features representing 1 day of data led to insufficient classifier accuracy, especially in the LR models, which only reached a maximum of 43% for the 3-class problem and 16% for the 5-class problem. The proposed hierarchical Bayesian LR method led to a significant increase in performance, reaching 64% accuracy, 0.81 AUC-ROC, and 0.70 AUC-PRC for the 3-class problem and 52% accuracy, 0.82 AUC-ROC, and 0.55 AUC-PRC for the 5-class problem. This demonstrates that accounting for individual differences through a simple hierarchical model can substantially improve emotional state prediction performance without relying on previous days of data.

### Feature Importance Analysis

**Figure 4:**
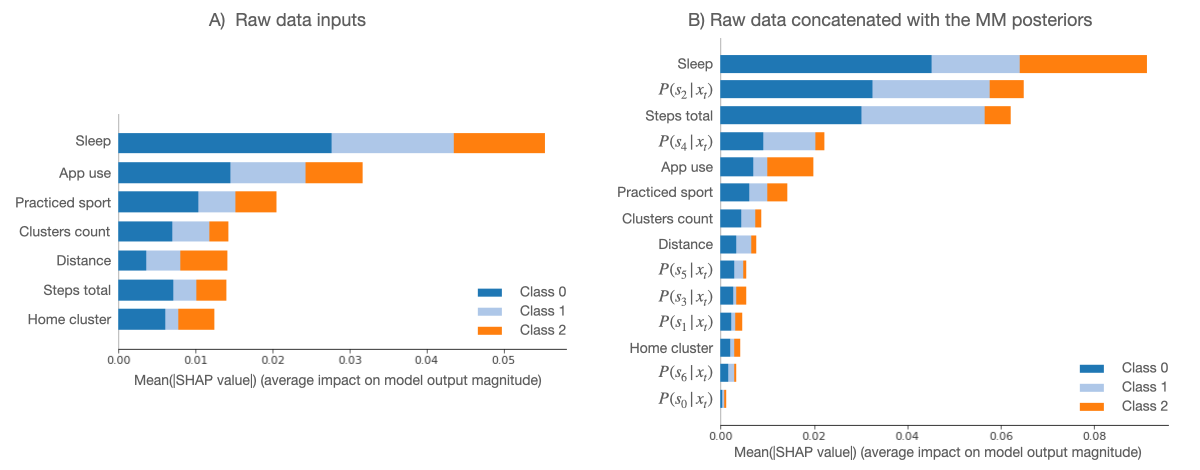
Summary plot of feature importance for the multilayer perceptron models for emotional valence prediction, showing raw data and raw data concatenated with mixture model posteriors. In terms of features, "steps total" refers to step count, "distance" refers to the distance traveled, "sleep" refers to the hours of sleep, "app use" refers to the hours spent using different apps, "home cluster" refers to the time spent at home, "clusters count" refers to the number of visited locations, "practiced sport" is an indicator of whether the patient practiced any sports, and "*P*(*s_i_* | *x_t_*)" refers to the posterior probability in component *i*. The following class labels were used: 0=negative; 1=neutral; and 2=positive emotional valence. MM: mixture model; SHAP: Shapley additive explanations.

Figure 4 provides an overview of which features are most important for the emotional valence MLP models using the raw data and using the raw data and MM posteriors as input features. To obtain an overview of which features are most important for the models, the mean SHAP values (*Evaluation* and *Interpretability* section) of every feature for every sample were computed. The plot below sorts features by the mean absolute value of the SHAP value magnitudes over all samples.

The hours of sleep and the time spent using their phone (app use) influenced all classes’ outcomes the most. The other features have an almost similar influence on the positive and negative classes. The negative output (class 0) is also strongly influenced by the step count, sport indicator, and time spent at home. If the posterior probabilities are used in combination with the raw features as inputs to the model, some outweigh the raw features in the decision-making process. For instance, the MLP relies heavily on the hours of sleep, the posterior probability of state 2, and the step count. The other classes seem to be more involved, requiring several posterior probabilities and raw values to form the prediction. The importance of posterior probabilities underlines the robust feature extraction provided by MM.

**Figure 5:**
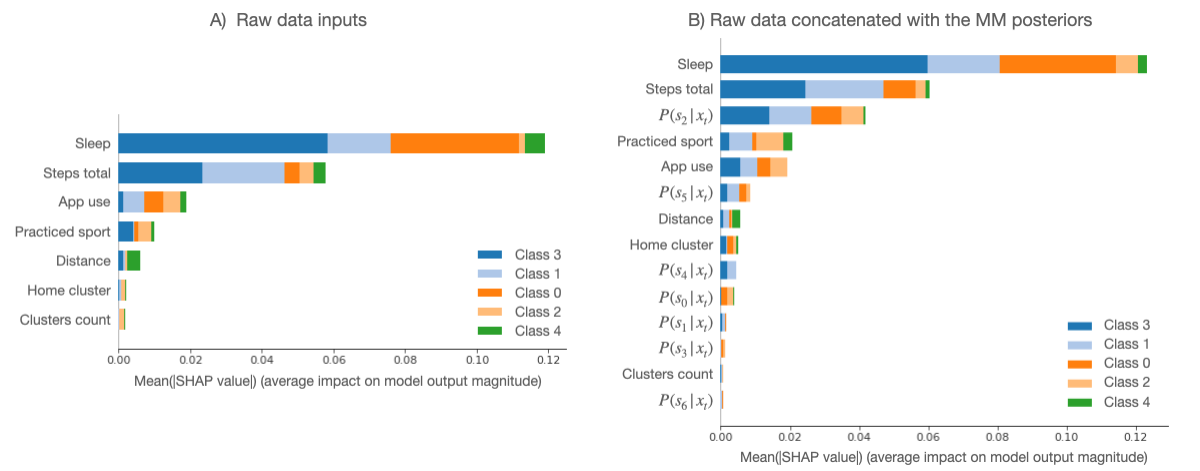
Summary plot of feature importance for the random forest models for emotional arousal-valence prediction, showing raw data and raw data concatenated with mixture model posteriors. In terms of features, "steps total" refers to step count, "distance" refers to the distance traveled, "sleep" refers to the hours of sleep, "app use" refers to the hours spent using different apps, "home cluster" refers to the time spent at home, "clusters count" refers to the number of visited locations, "practiced sport" is an indicator of whether the patient practiced any sports, and "*P*(*s_i_* | *x_t_*)" represents the posterior probability in component *i*. The following class labels were used: 0=neutral; 1=high arousal-positive valence; 2=high arousal-negative valence; 3=low arousal-negative valence; and 4=low arousal-positive valence. MM: mixture model; SHAP: Shapley additive explanations.

Similarly, the arousal-valence classifiers can be analyzed. In the raw data case (Figure 5), although the model emphasizes the hours of sleep and the step count, the other parameters become slightly less important. In the second case (Figure 5), some of the posterior probabilities seem to weigh more in the decision-making process than the raw features, as in the first experiment.

## Discussion

### Principal Findings

A variety of different machine learning methods were used to analyze passively sensed behavioral data from 6 sources (step count, distance traveled, hours of sleep, hours of phone use, time spent at home, number of locations visited, and a binary variable indicating whether the patient practiced sports during the day). These models were used to predict self-reported emotional state (valence or combination of valence and arousal) in a large, heterogeneous sample of treatment-seeking patients with clinically significant levels of psychological and/or emotional symptoms. Preliminary inspection of this data set revealed that the data exhibited significant missingness (approximately 84% [150615/179740] of the observations were partial). This represents real-world clinical data sets, which usually contain many missing samples and are sparsely labeled. The fact that this kind of data are both noisy and often nonrandomly missing means that the development of robust imputation techniques is a nontrivial problem. However, the development of such methods is vital if this type of information is used to support clinical decision making.

We addressed this problem by training generative models to handle missing data. These models were then used for data imputation and latent state (feature) extraction for emotional state prediction. Notably, predictive models performed significantly better when MM or HMM posterior probabilities were included alongside the raw behavioral input features. This suggests that the latent representation of the passively sensed behavioral variables discovered by the probabilistic generative models contains information relevant to daily emotional experience fluctuations. However, using HMMs over MMs did not improve the classification performance, which implies that there are no strong temporal correlations in the daily observations that can be captured by an HMM. Furthermore, in both experiments, the nonlinear models outperformed the other static models. The use of RNNs did not improve daily emotion predictions, suggesting that long-term behavior does not significantly influence patients’ everyday emotional states.

When using raw data alone as input features, the hours of sleep had the most substantial influence on the emotional state predictions. The importance of activity-related features varied between the 2 experimental set ups. When posterior state probabilities were included in the model, some proved to be more important than the raw features. This indicates that the MM provided excellent feature representation and filtering of the observed behavioral signals. Interestingly, an inspection of the confusion matrices for the best performing models revealed that, for the valence prediction analysis, models were more sensitive to the detection of negative, compared with positive or neutral emotional states. This is a useful feature as this is the domain of emotional experience most likely to be relevant for clinicians or self-monitoring of trends in overall mental health.

Finally, we proposed a hierarchical Bayesian regression with varying intercepts and a common slope to personalize the models. This approach performs personalized predictions while accounting for population-level characteristics. The personalized models using 1-day long feature vectors achieved similar performance to the nonlinear variants using 3-day long feature vectors. Moreover, they performed significantly better than global linear LR models. Personalized models outperforming the generalized models are intuitively reasonable as mood is very personal, and its perceptions among individuals differ.

### Limitations

This study has some limitations. As previously mentioned, the data analyzed in this study contained a large percentage of missing observations; approximately 84% (150,615/179,740) of the observations were partial, only a bit over 5% (9399/179,740) were complete, and the remaining 10% (19,726/179,740) were entirely missing. Some of the patient sequences had large chunks of consecutively missing observations, which could be because of sensor or software errors or the patients not using their devices for an extensive amount of time. Moreover, information about emotional states was sporadically reported. Therefore, only 10% (17,871/179,740) of the behavioral data were labeled with respect to the outcome of interest.

Recording emotions is a subjective process, and regular reflection of the emotional state may influence how one answers. The majority of the registered emotions were negatively valenced, meaning that the prediction models were somewhat biased toward negative emotional states. As a result, the models were most sensitive in the negative domain, and the overall prediction accuracies were not high in some cases. In addition, mood variability, another important point in psychiatric disorders, was not analyzed in this study. However, it will be important to explore in the future to better differentiate whether it is a pathological mood state or a mood within the normal range.

### Conclusions

This study is an initial step toward developing more robust and informed models for predicting emotional states from passively sensed data. It presents a sound basis for further exploration by proposing a solution to missing and sparsely labeled data, allowing the future focus to be directed toward developing more advanced models.

Future plans include examining other deep learning models to improve prediction accuracy and analyzing effects at a more refined time scale. Another intriguing question is to consider the effect of seasonality (weekdays and weekends, seasonal variation) on patients’ emotional states. Moreover, the possibilities of specialized models for different patient groups or individual patients will be further investigated.

## Appendix

Multimedia Appendix 1 Supplementary figures of the data missingness pattern and the mixture model and hidden Markov model structures for the emotional arousal-valence case.

Multimedia Appendix 2 An overview of the accuracy, the microaverage area under the receiver operating characteristics curve and area under the precision-recall curve scores, and confusion matrices for each of the models in the 3 experimental setups, as described in the Experiments section, for both the 3-class and 5-class experiments using the mixture model for missing data imputation and feature extraction.

## Abbreviations

AUC-PRC: area under the precision-recall curve
AUC-ROC: area under the receiver operating characteristics curve
Deep-DARWiN: Domain Alignment and Data Wrangling with Deep Generative Models
EMA: ecological momentary assessment
GRU: gated recurrent unit
HMM: hidden Markov model
LR: logistic regression
LSTM: long short-term memory
mHealth: mobile health
MLP: multilayer perceptron
MM: mixture model
RF: random forest
RFC: random forest classifier
RNN: recurrent neural network
SHAP: Shapley additive explanations
SVM: support vector machine
