# Supplementary material for: Predicting Emotional States Using Behavioral Markers Derived From Passively Sensed Data: Data-Driven Machine Learning Approach
Source: JMIR Mhealth Uhealth. 2021 Mar 22;9(3):e24465. doi: 10.2196/24465 (PMC8088855; doi:10.2196/24465)

Multimedia Appendix 1

Figure S1 - The distribution of missing data in the selected observation sequences.

Black indicates the presence of observations, and white the lack of them. Feature notations: steps total = step count, distance = distance travelled, sleep = hours of sleep, app use = hours spent using different apps, home cluster = time spent at home, clusters count = number of visited locations, practiced sport = indicator whether the patient practiced any sports, emotion = emotional state (valence or arousal-valence).

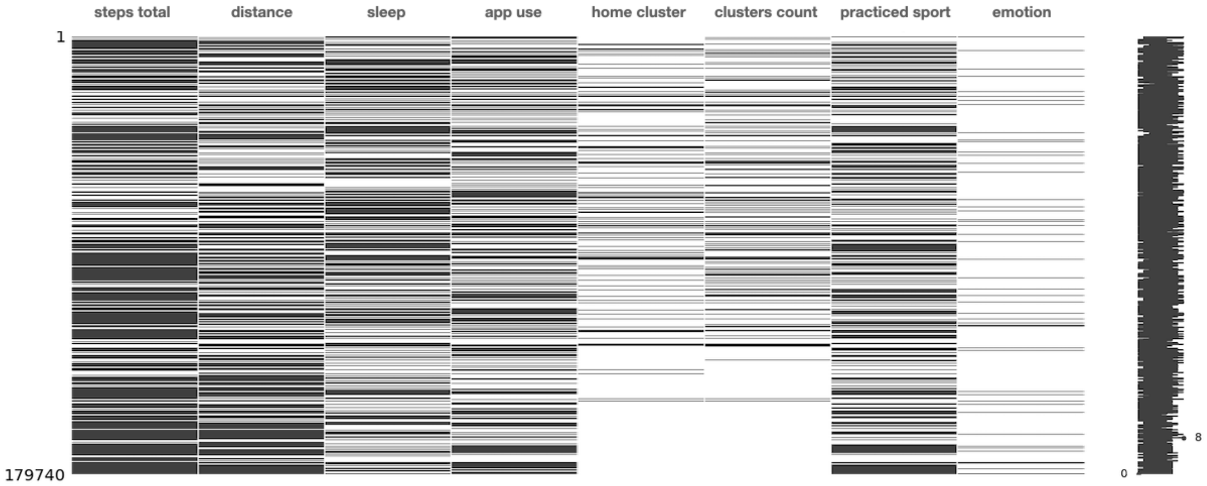

Figure S2 - The structure of the 7-component MM used for emotional arousal-valence modelling.

The structure of the 7-component MM used for emotional arousal-valence modelling with the means of each Gaussian in each component and indicated discrete emission probabilities. The size of the icons indicates the magnitude of the discrete emission probabilities (emotion and sport). Note that the negative mean values resulted because of the normalisation of the features. Abbreviations: LA-NV = low arousal-negative valence, HA-NV = high arousal-negative valence, HA-PV = high arousal-positive valence, LA-PV = low arousal-positive valence. Feature notations: steps total = step count, distance = distance travelled, sleep = hours of sleep, app use = hours spent using different apps, home cluster = time spent at home, clusters count = number of visited locations, practiced sport = indicator whether the patient practiced any sports. Similarly to the emotional valence case, the MM components capture different behaviours.

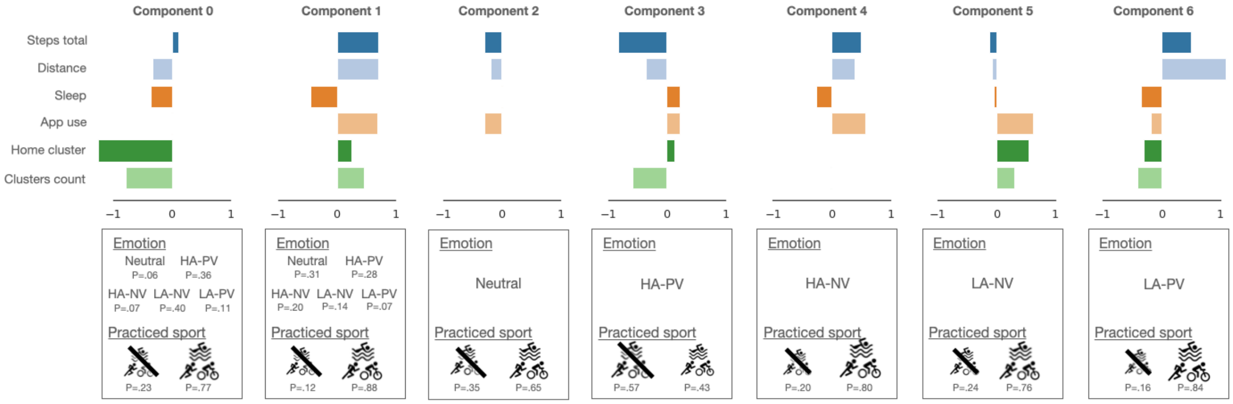

Figure S3 - The structure of the 7-state HMM used for emotional arousal-valence modelling.

The structure of the 7-state HMM used for emotional arousal - valence modelling with the means of each Gaussian in each state and indicated discrete emission probabilities. Only the transitions with higher than 0.1 probability are shown in the graph. Note that the negative mean values resulted because of the normalisation of the features. Abbreviations: LA-NV = low arousal-negative valence, HA-NV = high arousal-negative valence, HA-PV = high arousal-positive valence, LA-PV = low arousal-positive valence. Feature notations: steps total = step count, distance = distance travelled, sleep = hours of sleep, app use = hours spent using different apps, home cluster = time spent at home, clusters count = number of visited locations, practiced sport = indicator whether the patient practiced any sports.

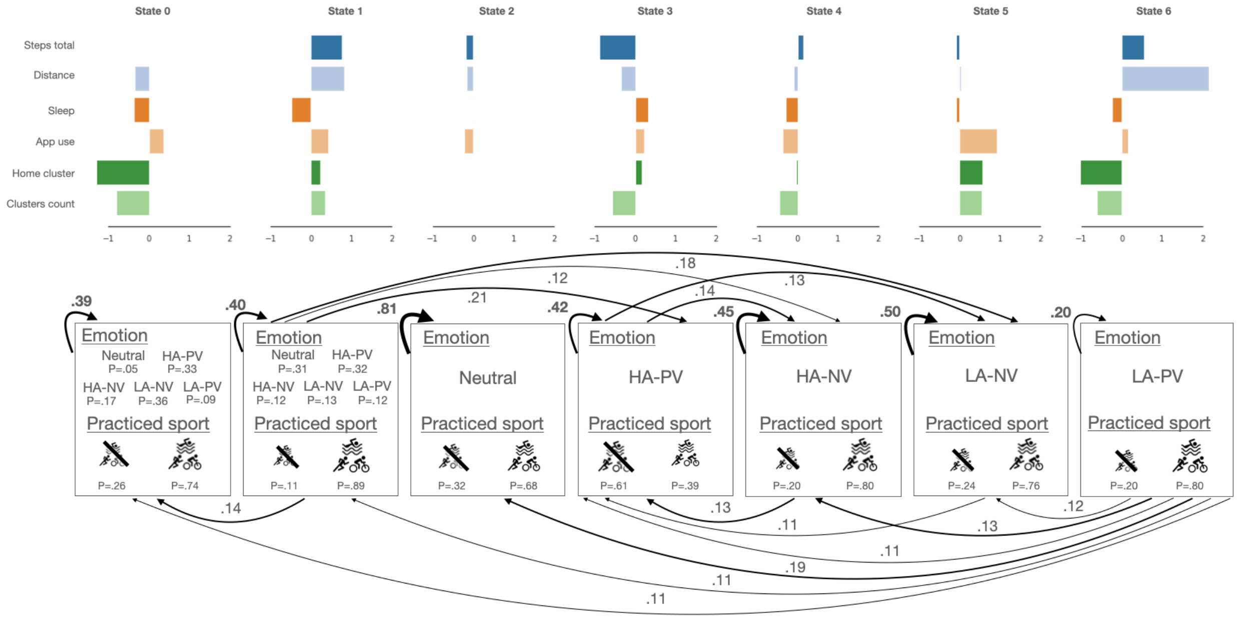

Supplement: Multimedia Appendix 1 [file mhealth_v9i3e24465_app1.pdf]
