# Supplementary material for: Predicting Emotional States Using Behavioral Markers Derived From Passively Sensed Data: Data-Driven Machine Learning Approach
Source: JMIR Mhealth Uhealth. 2021 Mar 22;9(3):e24465. doi: 10.2196/24465 (PMC8088855; doi:10.2196/24465)
Supplement: Multimedia Appendix 2 [file mhealth_v9i3e24465_app2.pdf]

Multimedia Appendix 2

Model notations: LR/SVC/RFC/MLP - x = LR/SVC/RFC/MLP classifiers trained with input features formed of x-days of observations concatenated to create a single feature vector. RNN/LSTM/GRU - x = RNN/LSTM/GRU - RNNs with different cells using x-months long input sequences. Input feature notations: w/o posteriors = raw features used as classifier input; only posteriors = the MM component posterior probabilities used as classifier input features; w/ posteriors = raw features concatenated with the MM component posterior probabilities used as classifier input features. Model abbreviations: LR = logistic regression, SVC = support vector classifier, RFC = random forest classifier, MLP = multilayer perceptron, RNN = recurrent neural network, LSTM = long short term memory, GRU = gated reccurent unit.

Figure S1 - Classifier performance comparison plot - Emotional valence case.

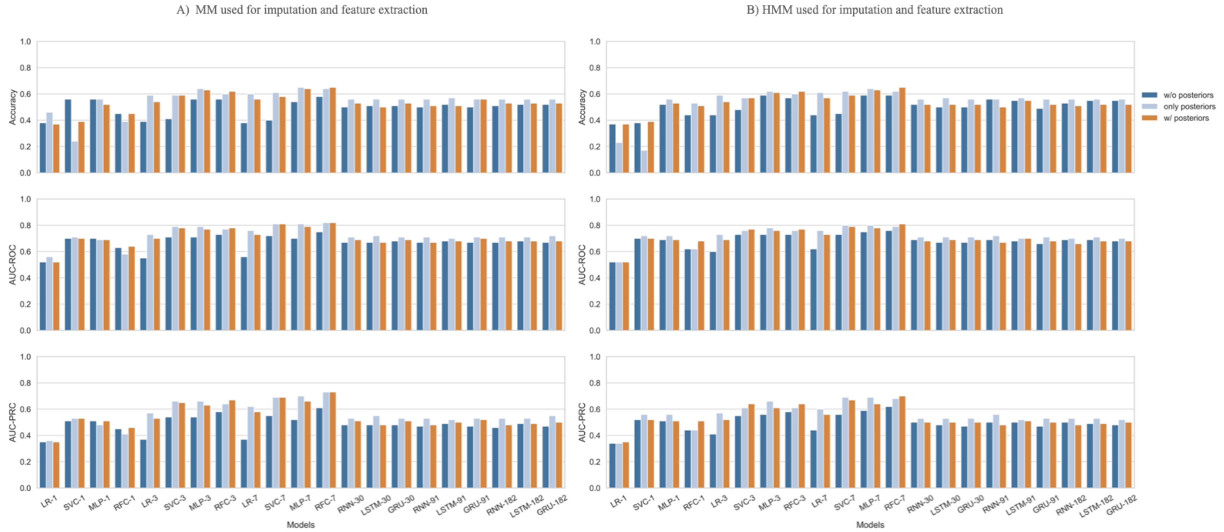

Table S1 - Classifier performance overview - Emotional valence case.

Class labels: 0 = negative, 1 = neutral, 2 = positive emotional valence.

Model	Input Features	Accuracy	AUC-ROC	AUC-PRC	Confusion Matrix
LR-1	w/o posteriors	0.38	0.52	0.35	0 1 2<br>0: 690 596 557<br>1: 201 177 176<br>2: 340 176 367
	w/ posteriors	0.37	0.52	0.35	0 1 2<br>0: 692 597 554<br>1: 201 175 178<br>2: 338 185 360
	only posteriors	0.46	0.56	0.36	0 1 2<br>0: 1038 0 805<br>1: 296 0 258<br>2: 406 0 477
LR-3	w/o posteriors	0.39	0.55	0.37	0 1 2<br>0: 622 687 500<br>1: 188 170 175<br>2: 238 170 450
	w/ posteriors	0.54	0.7	0.53	0 1 2<br>0: 1050 467 292<br>1: 173 196 164<br>2: 197 181 480

Table S1 continued from previous page

Model	Input Features	Accuracy	AUC-ROC	AUC-PRC	Confusion Matrix
	only posteriors	0.59	0.73	0.57	0 1 2<br>0 | 1235 297 277<br>1 | 213 147 173<br>2 | 234 131 493
LR-7	w/o posteriors	0.38	0.56	0.37	0 1 2<br>0 | 592 699 458<br>1 | 185 149 168<br>2 | 233 155 430
	w/ posteriors	0.56	0.73	0.58	0 1 2<br>0 | 1074 403 272<br>1 | 158 193 151<br>2 | 180 176 462
	only posteriors	0.6	0.76	0.62	0 1 2<br>0 | 1226 275 248<br>1 | 192 159 151<br>2 | 211 149 458
MLP-1	w/o posteriors	0.56	0.7	0.51	0 1 2<br>0 | 1837 4 2<br>1 | 554 0 0<br>2 | 878 2 3
	w/ posteriors	0.52	0.69	0.51	0 1 2<br>0 | 1572 110 161<br>1 | 478 30 46<br>2 | 741 27 115
	only posteriors	0.56	0.69	0.48	0 1 2<br>0 | 1843 0 0<br>1 | 554 0 0<br>2 | 883 0 0
MLP-3	w/o posteriors	0.56	0.71	0.54	0 1 2<br>0 | 1474 188 147<br>1 | 414 43 76<br>2 | 557 38 263
	w/ posteriors	0.63	0.77	0.63	0 1 2<br>0 | 1515 102 192<br>1 | 333 82 118<br>2 | 403 51 404
	only posteriors	0.64	0.79	0.66	0 1 2<br>0 | 1595 33 181<br>1 | 358 44 131<br>2 | 439 14 405
MLP-7	w/o posteriors	0.54	0.7	0.52	0 1 2<br>0 | 1418 97 234<br>1 | 397 27 78<br>2 | 564 35 219
	w/ posteriors	0.64	0.79	0.66	0 1 2<br>0 | 1474 96 179<br>1 | 310 88 104<br>2 | 369 61 388
	only posteriors	0.65	0.81	0.7	0 1 2<br>0 | 1533 51 165<br>1 | 307 66 129<br>2 | 368 45 405

Table S1 continued from previous page

Model	Input Features	Accuracy	AUC-ROC	AUC-PRC	Confusion Matrix
RFC-1	w/o posteriors	0.45	0.63	0.45	0 1 2<br>0 | 1072 350 421<br>1 | 306 99 149<br>2 | 461 128 294
	w/ posteriors	0.45	0.64	0.46	0 1 2<br>0 | 1097 314 432<br>1 | 320 68 166<br>2 | 472 98 313
	only posteriors	0.39	0.58	0.41	0 1 2<br>0 | 856 421 566<br>1 | 251 119 184<br>2 | 409 170 304
RFC-3	w/o posteriors	0.56	0.73	0.58	0 1 2<br>0 | 1240 257 312<br>1 | 287 85 161<br>2 | 307 70 481
	w/ posteriors	0.62	0.78	0.67	0 1 2<br>0 | 1355 160 294<br>1 | 246 101 186<br>2 | 259 67 532
	only posteriors	0.6	0.77	0.64	0 1 2<br>0 | 1258 213 338<br>1 | 190 137 206<br>2 | 221 113 524
RFC-7	w/o posteriors	0.58	0.75	0.61	0 1 2<br>0 | 1256 211 282<br>1 | 283 55 164<br>2 | 290 50 478
	w/ posteriors	0.65	0.82	0.73	0 1 2<br>0 | 1369 76 304<br>1 | 228 89 185<br>2 | 238 50 530
	only posteriors	0.64	0.82	0.73	0 1 2<br>0 | 1327 107 315<br>1 | 194 99 209<br>2 | 196 73 549
SVC-1	w/o posteriors	0.56	0.7	0.51	0 1 2<br>0 | 1837 4 2<br>1 | 554 0 0<br>2 | 878 2 3
	w/ posteriors	0.39	0.7	0.53	0 1 2<br>0 | 775 563 505<br>1 | 221 149 184<br>2 | 378 156 349
	only posteriors	0.24	0.71	0.53	0 1 2<br>0 | 0 1048 795<br>1 | 0 297 257<br>2 | 0 409 474
SVC-3	w/o posteriors	0.41	0.71	0.54	0 1 2<br>0 | 714 616 479<br>1 | 222 156 155<br>2 | 267 162 429

Table S1 continued from previous page

Model	Input Features	Accuracy	AUC-ROC	AUC-PRC	Confusion Matrix
	w/ posteriors	0.59	0.78	0.65	0 1 2<br>0: 1271 286 252<br>1: 231 157 145<br>2: 276 132 450
	only posteriors	0.59	0.79	0.66	0 1 2<br>0: 1259 285 265<br>1: 225 146 162<br>2: 253 124 481
SVC-7	w/o posteriors	0.4	0.72	0.55	0 1 2<br>0: 679 634 436<br>1: 198 126 178<br>2: 237 150 431
	w/ posteriors	0.58	0.81	0.69	0 1 2<br>0: 1170 321 258<br>1: 176 169 157<br>2: 196 166 456
	only posteriors	0.61	0.81	0.69	0 1 2<br>0: 1274 239 236<br>1: 203 144 155<br>2: 235 122 461
RNN-30	w/o posteriors	0.5	0.67	0.48	0 1 2<br>0: 1283 142 263<br>1: 387 31 80<br>2: 597 32 185
	w/ posteriors	0.53	0.69	0.51	0 1 2<br>0: 1468 57 163<br>1: 432 12 54<br>2: 687 10 117
	only posteriors	0.56	0.71	0.53	0 1 2<br>0: 1661 0 27<br>1: 490 0 8<br>2: 789 0 25
RNN-91	w/o posteriors	0.5	0.67	0.47	0 1 2<br>0: 1411 150 188<br>1: 449 31 54<br>2: 684 25 132
	w/ posteriors	0.51	0.67	0.48	0 1 2<br>0: 1440 170 139<br>1: 459 33 42<br>2: 688 25 128
	only posteriors	0.56	0.71	0.53	0 1 2<br>0: 1749 0 0<br>1: 534 0 0<br>2: 841 0 0
RNN-182	w/o posteriors	0.51	0.67	0.46	0 1 2<br>0: 1382 115 279<br>1: 419 33 84<br>2: 637 27 187
	w/ posteriors	0.53	0.68	0.48	0 1 2<br>0: 1508 139 129<br>1: 459 32 45<br>2: 707 23 121

Table S1 continued from previous page

Model	Input Features	Accuracy	AUC-ROC	AUC-PRC	Confusion Matrix
	only posteriors	0.56	0.71	0.53	$\begin{array}{c\|ccc} & 0 & 1 & 2 \\ \hline 0 & 1705 & 0 & 71 \\ 1 & 512 & 0 & 24 \\ 2 & 770 & 0 & 81 \end{array}$
LSTM-30	w/o posteriors	0.51	0.67	0.48	$\begin{array}{c\|ccc} & 0 & 1 & 2 \\ \hline 0 & 1336 & 139 & 213 \\ 1 & 401 & 30 & 67 \\ 2 & 626 & 27 & 161 \end{array}$
	w/ posteriors	0.5	0.67	0.48	$\begin{array}{c\|ccc} & 0 & 1 & 2 \\ \hline 0 & 1303 & 146 & 239 \\ 1 & 408 & 17 & 73 \\ 2 & 585 & 44 & 185 \end{array}$
	only posteriors	0.56	0.72	0.55	$\begin{array}{c\|ccc} & 0 & 1 & 2 \\ \hline 0 & 1688 & 0 & 0 \\ 1 & 498 & 0 & 0 \\ 2 & 814 & 0 & 0 \end{array}$
LSTM-91	w/o posteriors	0.52	0.68	0.49	$\begin{array}{c\|ccc} & 0 & 1 & 2 \\ \hline 0 & 1483 & 82 & 184 \\ 1 & 444 & 28 & 62 \\ 2 & 709 & 18 & 114 \end{array}$
	w/ posteriors	0.51	0.68	0.5	$\begin{array}{c\|ccc} & 0 & 1 & 2 \\ \hline 0 & 1418 & 134 & 197 \\ 1 & 441 & 31 & 62 \\ 2 & 681 & 18 & 142 \end{array}$
	only posteriors	0.57	0.7	0.52	$\begin{array}{c\|ccc} & 0 & 1 & 2 \\ \hline 0 & 1669 & 0 & 80 \\ 1 & 492 & 0 & 42 \\ 2 & 729 & 0 & 112 \end{array}$
LSTM-182	w/o posteriors	0.52	0.68	0.49	$\begin{array}{c\|ccc} & 0 & 1 & 2 \\ \hline 0 & 1520 & 100 & 156 \\ 1 & 470 & 31 & 35 \\ 2 & 750 & 22 & 79 \end{array}$
	w/ posteriors	0.53	0.68	0.49	$\begin{array}{c\|ccc} & 0 & 1 & 2 \\ \hline 0 & 1583 & 71 & 122 \\ 1 & 487 & 25 & 24 \\ 2 & 756 & 21 & 74 \end{array}$
	only posteriors	0.56	0.71	0.53	$\begin{array}{c\|ccc} & 0 & 1 & 2 \\ \hline 0 & 1776 & 0 & 0 \\ 1 & 536 & 0 & 0 \\ 2 & 851 & 0 & 0 \end{array}$
GRU-30	w/o posteriors	0.51	0.68	0.48	$\begin{array}{c\|ccc} & 0 & 1 & 2 \\ \hline 0 & 1363 & 114 & 211 \\ 1 & 401 & 32 & 65 \\ 2 & 649 & 24 & 141 \end{array}$
	w/ posteriors	0.53	0.69	0.51	$\begin{array}{c\|ccc} & 0 & 1 & 2 \\ \hline 0 & 1379 & 77 & 232 \\ 1 & 418 & 17 & 63 \\ 2 & 613 & 18 & 183 \end{array}$
	only posteriors	0.56	0.71	0.53	$\begin{array}{c\|ccc} & 0 & 1 & 2 \\ \hline 0 & 1663 & 0 & 25 \\ 1 & 490 & 0 & 8 \\ 2 & 791 & 0 & 23 \end{array}$

Table S1 continued from previous page

Model	Input Features	Accuracy	AUC-ROC	AUC-PRC	Confusion Matrix	0	1	2
GRU-91	w/o posteriors	0.5	0.67	0.47	0	1414	144	191
					1	441	35	58
					2	689	36	116
	w/ posteriors	0.56	0.7	0.52	0	1742	3	4
					1	531	1	2
					2	826	0	15
	only posteriors	0.56	0.71	0.53	0	1743	1	5
					1	533	0	1
					2	840	0	1
GRU-182	w/o posteriors	0.52	0.67	0.47	0	1518	93	165
					1	465	28	43
					2	726	37	88
	w/ posteriors	0.53	0.68	0.5	0	1527	94	155
					1	467	30	39
					2	707	34	110
	only posteriors	0.56	0.72	0.55	0	1774	0	2
					1	535	0	1
					2	851	0	0

Table S2 - Classifier performance overview - Emotional arousal-valence case.

Class labels: 0 - neutral, 1 - high arousal - positive valence, 2 - high arousal - negative valence, 3 - low arousal - negative valence, 4 - low arousal - positive valence.

Model	Input Features	Accuracy	AUC-ROC	AUC-PRC	Confusion Matrix	0	1	2	3	4
					0	155	165	6	181	47
LR-1	w/o posteriors	0.27	0.52	0.22	1	124	271	5	206	47
					2	150	138	0	168	24
					3	431	397	2	438	95
					4	48	74	0	76	32
					0	155	165	6	181	47
	w/ posteriors	0.27	0.52	0.22	1	124	271	5	206	47
					2	150	138	0	168	24
					3	431	397	2	438	95
					4	48	74	0	76	32
					0	314	123	0	13	104
	only posteriors	0.16	0.54	0.21	1	308	139	0	26	180
					2	292	81	0	19	88
					3	798	260	0	41	264
					4	114	67	0	10	39
					0	177	134	27	87	108
LR-3	w/o posteriors	0.27	0.53	0.22	1	111	240	22	134	123
					2	158	129	19	98	66
					3	422	337	43	349	188
					4	45	58	9	50	66
					0	206	122	68	80	57
	w/ posteriors	0.29	0.56	0.24	1	129	230	50	140	81
					2	117	108	128	82	35
					3	338	334	218	311	138
					4	61	65	16	36	50
					0	174	69	81	96	113
	only posteriors	0.34	0.63	0.29	1	99	142	63	154	172
					2	90	55	155	108	62
					3	225	132	283	540	159
					4	49	28	19	55	77
					0	151	120	15	121	95
LR-7	w/o posteriors	0.26	0.53	0.22	1	99	237	19	148	92
					2	153	126	19	104	53
					3	427	343	50	321	153
					4	51	59	5	51	57
					0	183	98	55	78	88
	w/ posteriors	0.28	0.56	0.24	1	109	211	34	142	99
					2	115	85	133	66	56
					3	340	288	218	268	180
					4	52	59	16	40	56
					0	169	78	72	78	105
	only posteriors	0.36	0.65	0.31	1	96	185	55	108	151
					2	82	54	163	102	54
					3	210	107	291	504	182
					4	46	27	16	45	89

Table S2 continued from previous page

Model	Input Features	Accuracy	AUC-ROC	AUC-PRC	Confusion Matrix	0	1	2	3	4
MLP-1	w/o posteriors	0.37	0.69	0.34	0	41	47	0	465	1
					1	23	81	0	549	0
					2	51	46	0	383	0
					3	156	101	0	1105	1
					4	20	14	0	196	0
	w/ posteriors	0.38	0.69	0.35	0	29	85	0	440	0
					1	15	148	0	490	0
					2	37	75	0	368	0
					3	99	191	0	1073	0
					4	15	26	0	189	0
	only posteriors	0.42	0.69	0.35	0	0	28	0	526	0
					1	0	56	0	597	0
					2	0	21	0	459	0
					3	0	45	0	1318	0
					4	0	17	0	213	0
MLP-3	w/o posteriors	0.36	0.68	0.33	0	77	89	2	359	6
					1	41	169	9	402	9
					2	63	99	2	304	2
					3	211	216	6	887	19
					4	29	28	1	169	1
	w/ posteriors	0.4	0.72	0.4	0	91	108	2	332	0
					1	54	200	2	372	2
					2	50	88	19	313	0
					3	148	192	14	984	1
					4	29	50	2	147	0
	only posteriors	0.44	0.73	0.42	0	130	81	12	310	0
					1	69	159	12	390	0
					2	55	42	30	343	0
					3	134	93	38	1074	0
					4	39	35	0	154	0
MLP-7	w/o posteriors	0.34	0.66	0.3	0	94	75	0	332	1
					1	37	142	0	412	4
					2	88	74	0	293	0
					3	317	175	0	798	4
					4	40	22	0	161	0
	w/ posteriors	0.41	0.72	0.4	0	74	122	19	278	9
					1	46	258	6	284	1
					2	48	80	52	275	0
					3	158	163	87	870	16
					4	32	59	1	128	3
	only posteriors	0.46	0.74	0.45	0	74	70	3	355	0
					1	43	160	0	392	0
					2	26	35	0	394	0
					3	56	54	1	1183	0
					4	12	27	0	184	0

Table S2 continued from previous page

Model	Input Features	Accuracy	AUC-ROC	AUC-PRC	Confusion Matrix					
						0	1	2	3	4
					0	127	149	41	227	10
RFC-1	w/o posteriors	0.29	0.62	0.27	1	119	220	53	236	25
					2	113	123	35	195	14
					3	374	290	96	543	60
					4	53	55	10	100	12
						0	1	2	3	4
					0	126	145	31	245	7
	w/ posteriors	0.3	0.63	0.28	1	116	209	51	261	16
					2	108	112	32	213	15
					3	364	282	81	592	44
					4	52	54	11	98	15
						0	1	2	3	4
					0	313	98	41	74	28
	only posteriors	0.19	0.57	0.23	1	331	119	65	85	53
					2	291	62	24	71	32
					3	791	244	89	172	67
					4	120	43	10	46	11
						0	1	2	3	4
					0	156	154	43	158	22
RFC-3	w/o posteriors	0.33	0.66	0.3	1	73	269	35	224	29
					2	89	126	58	185	12
					3	304	327	110	554	44
					4	48	82	7	74	17
						0	1	2	3	4
					0	126	133	50	140	84
	w/ posteriors	0.44	0.74	0.44	1	72	394	33	86	45
					2	61	92	86	214	17
					3	169	212	146	735	77
					4	37	55	17	52	67
						0	1	2	3	4
					0	140	127	63	88	115
	only posteriors	0.41	0.71	0.4	1	90	351	52	47	90
					2	67	89	129	147	38
					3	179	200	242	600	118
					4	48	38	19	39	84
						0	1	2	3	4
					0	131	134	13	214	10
RFC-7	w/o posteriors	0.35	0.67	0.33	1	58	228	8	288	13
					2	85	95	27	243	5
					3	305	250	46	666	27
					4	55	56	1	101	10
						0	1	2	3	4
					0	116	156	18	148	64
	w/ posteriors	0.48	0.77	0.5	1	56	402	17	95	25
					2	50	89	67	232	17
					3	128	186	91	820	69
					4	33	59	5	64	62
						0	1	2	3	4
					0	107	143	38	125	89
	only posteriors	0.46	0.77	0.49	1	60	382	26	73	54
					2	42	91	90	209	23
					3	116	168	160	756	94
					4	28	46	6	56	87

Table S2 continued from previous page

Model	Input Features	Accuracy	AUC-ROC	AUC-PRC	Confusion Matrix					
						0	1	2	3	4
					0	179	102	7	251	15
SVC-1	w/o posteriors	0.28	0.69	0.35	1	174	138	23	295	23
					2	177	76	6	212	9
					3	505	203	18	600	37
					4	66	38	10	106	10
						0	1	2	3	4
					0	175	98	20	238	23
	w/ posteriors	0.28	0.69	0.35	1	187	138	14	274	40
					2	176	74	6	205	19
					3	499	198	23	582	61
					4	68	39	6	105	12
						0	1	2	3	4
					0	314	76	0	0	164
	only posteriors	0.14	0.69	0.35	1	308	55	0	0	290
					2	292	53	0	0	135
					3	798	168	0	0	397
					4	114	36	0	0	80
						0	1	2	3	4
					0	148	125	78	144	38
SVC-3	w/o posteriors	0.28	0.69	0.35	1	96	212	62	194	66
					2	135	117	49	137	32
					3	384	297	127	448	83
					4	44	53	27	73	31
						0	1	2	3	4
					0	200	103	54	110	66
	w/ posteriors	0.31	0.71	0.38	1	123	207	33	176	91
					2	104	92	102	123	49
					3	317	243	175	443	161
					4	58	52	23	55	40
						0	1	2	3	4
					0	192	45	80	125	91
	only posteriors	0.35	0.72	0.42	1	112	88	60	209	161
					2	99	31	144	145	51
					3	237	56	267	632	147
					4	58	21	17	72	60
						0	1	2	3	4
					0	156	95	45	149	57
SVC-7	w/o posteriors	0.27	0.68	0.34	1	111	187	50	187	60
					2	161	93	47	124	30
					3	428	264	105	411	86
					4	60	47	23	64	29
						0	1	2	3	4
					0	176	112	39	100	75
	w/ posteriors	0.3	0.71	0.39	1	112	202	22	148	111
					2	103	86	97	106	63
					3	280	246	165	392	211
					4	62	53	11	55	42
						0	1	2	3	4
					0	151	102	71	78	100
	only posteriors	0.36	0.75	0.45	1	83	195	54	131	132
					2	78	51	158	111	57
					3	189	129	287	503	186
					4	37	35	14	52	85

Table S2 continued from previous page

Model	Input Features	Accuracy	AUC-ROC	AUC-PRC	Confusion Matrix					
						0	1	2	3	4
					0	5	53	0	440	0
RNN-30	w/o posteriors	0.4	0.69	0.35	1	12	77	0	530	0
					2	3	50	0	395	0
					3	4	114	0	1122	0
					4	1	17	0	177	0
						0	1	2	3	4
					0	50	93	0	355	0
	w/ posteriors	0.36	0.67	0.31	1	11	177	0	431	0
					2	68	75	0	305	0
					3	200	195	0	840	5
					4	28	33	0	131	3
						0	1	2	3	4
					0	0	0	0	498	0
	only posteriors	0.41	0.69	0.36	1	0	0	0	619	0
					2	0	0	0	448	0
					3	0	0	0	1240	0
					4	0	0	0	195	0
						0	1	2	3	4
					0	35	87	0	397	15
RNN-91	w/o posteriors	0.35	0.67	0.31	1	14	173	0	434	8
					2	86	66	0	311	2
					3	197	171	0	857	59
					4	14	29	0	156	13
						0	1	2	3	4
					0	38	85	0	403	8
	w/ posteriors	0.36	0.68	0.32	1	19	172	1	435	2
					2	60	64	0	340	1
					3	163	181	0	894	46
					4	14	24	0	164	10
						0	1	2	3	4
					0	5	22	0	507	0
	only posteriors	0.42	0.7	0.35	1	1	52	0	576	0
					2	3	10	0	452	0
					3	3	24	0	1257	0
					4	0	5	0	207	0
						0	1	2	3	4
					0	43	42	0	451	0
RNN-182	w/o posteriors	0.36	0.67	0.32	1	36	74	0	518	1
					2	33	48	0	385	1
					3	154	120	0	1035	0
					4	23	17	0	182	0
						0	1	2	3	4
					0	35	54	3	444	0
	w/ posteriors	0.39	0.67	0.33	1	17	114	8	489	1
					2	32	53	0	382	0
					3	111	124	0	1074	0
					4	17	16	0	189	0
						0	1	2	3	4
					0	0	22	0	514	0
	only posteriors	0.42	0.69	0.35	1	0	53	0	576	0
					2	0	10	0	457	0
					3	0	25	0	1284	0
					4	0	3	0	219	0

Table S2 continued from previous page

Model	Input Features	Accuracy	AUC-ROC	AUC-PRC	Confusion Matrix	0	1	2	3	4
LSTM-30	w/o posteriors	0.33	0.67	0.3	0	59	75	0	361	3
					1	55	136	0	426	2
					2	82	69	0	296	1
					3	257	184	0	780	19
					4	30	32	0	131	2
	w/ posteriors	0.36	0.67	0.32	0	50	84	0	362	2
					1	39	154	0	424	2
					2	69	62	0	317	0
					3	211	162	0	861	6
					4	28	32	0	134	1
	only posteriors	0.42	0.69	0.37	0	0	4	0	494	0
					1	0	31	0	588	0
					2	0	1	0	447	0
					3	0	10	0	1230	0
					4	0	1	0	194	0
LSTM-91	w/o posteriors	0.33	0.68	0.32	0	50	67	0	415	2
					1	38	126	0	459	6
					2	71	52	0	341	1
					3	242	171	0	868	3
					4	29	27	0	156	0
	w/ posteriors	0.33	0.67	0.32	0	62	80	0	389	3
					1	51	174	0	400	4
					2	85	67	0	312	1
					3	265	217	0	790	12
					4	28	27	0	156	1
	only posteriors	0.41	0.69	0.35	0	0	5	0	529	0
					1	0	9	0	620	0
					2	0	0	0	465	0
					3	0	2	0	1282	0
					4	0	0	0	212	0
LSTM-182	w/o posteriors	0.35	0.65	0.3	0	50	80	0	399	7
					1	23	149	3	443	11
					2	46	70	0	347	4
					3	177	197	0	905	30
					4	27	26	1	163	5
	w/ posteriors	0.36	0.68	0.33	0	50	19	0	467	0
					1	43	59	0	527	0
					2	36	26	0	405	0
					3	182	90	0	1037	0
					4	28	6	0	188	0
	only posteriors	0.42	0.69	0.37	0	0	22	0	514	0
					1	0	51	0	578	0
					2	0	12	0	455	0
					3	0	19	0	1290	0
					4	0	8	0	214	0

Table S2 continued from previous page

Model	Input Features	Accuracy	AUC-ROC	AUC-PRC	Confusion Matrix					
						0	1	2	3	4
					0	54	92	0	349	3
					1	32	156	0	428	3
GRU-30	w/o posteriors	0.34	0.67	0.31	2	70	79	0	297	2
					3	211	213	0	793	23
					4	29	39	0	123	4
						0	1	2	3	4
					0	44	93	4	350	7
					1	18	175	1	418	7
	w/ posteriors	0.34	0.67	0.31	2	74	69	0	300	5
					3	201	187	1	810	41
					4	29	37	0	124	5
						0	1	2	3	4
					0	0	22	0	476	0
					1	0	59	0	560	0
	only posteriors	0.42	0.69	0.36	2	0	13	0	434	1
					3	0	29	0	1211	0
					4	0	8	0	187	0
						0	1	2	3	4
					0	74	63	0	396	1
					1	60	107	0	459	3
GRU-91	w/o posteriors	0.33	0.67	0.32	2	87	54	0	324	0
					3	279	152	0	851	2
					4	31	23	0	158	0
						0	1	2	3	4
					0	56	73	0	399	6
					1	35	184	0	408	2
	w/ posteriors	0.34	0.67	0.32	2	75	55	0	330	5
					3	253	182	0	819	30
					4	29	26	0	152	5
						0	1	2	3	4
					0	6	1	0	527	0
					1	9	2	0	618	0
	only posteriors	0.41	0.69	0.35	2	5	0	0	460	0
					3	8	1	0	1275	0
					4	1	0	0	211	0
						0	1	2	3	4
					0	57	70	0	406	3
					1	40	138	0	451	0
GRU-182	w/o posteriors	0.33	0.65	0.29	2	77	60	0	326	4
					3	243	174	0	856	36
					4	24	25	0	172	1
						0	1	2	3	4
					0	56	36	0	444	0
					1	50	96	0	483	0
	w/ posteriors	0.36	0.67	0.33	2	41	42	0	384	0
					3	211	117	0	975	6
					4	28	8	0	186	0
						0	1	2	3	4
					0	0	0	0	536	0
					1	0	0	0	629	0
	only posteriors	0.41	0.68	0.35	2	0	0	0	467	0
					3	0	0	0	1309	0
					4	0	0	0	222	0
